# De-novo multilayering in fibrovascular pigment epithelial detachment

**DOI:** 10.1038/s41598-021-96746-1

**Published:** 2021-08-26

**Authors:** Manoj Soman, Jay U. Sheth, Asmita Indurkar, Padmanabhan Meleth, Unnikrishnan Nair

**Affiliations:** 1 Vitreoretinal Services, Chaithanya Eye Hospital and Research Institute, Trivandrum, India; 2 Chaithanya Innovation in Technology and Eyecare (Research), Trivandrum, India

**Keywords:** Anatomy, Biomarkers, Medical research, Pathogenesis

## Abstract

This study describes the occurrence of multilayered pigment-epithelial detachment (MLPED) as a De-novo phenomenon (DN-MLPED) and compare the features with multi-layering secondary to chronic anti-vascular endothelial growth factor (anti-VEGF) therapy (s-MLPED). We did a retrospective evaluation of spectral-domain optical coherence tomography (SD-OCT) features, treatment-profile, and visual-acuity (VA) outcomes in eyes with MLPED. Out of 17 eyes with MLPED, 7 eyes had DN-MLPED and 10 eyes had s-MLPED. There was no significant difference in baseline and final VA between the groups. At the final visit, no significant visual improvement was noted in both the groups, although a possible trend towards an improvement was seen in DN-MLPED eyes while the s-MLPED demonstrated a declining trend (DN-MLPED—LogMAR-BCVA: Baseline = 0.79 [∼ 20/123] ± 0.91; Final = 0.76 [∼ 20/115] ± 0.73; *p* = 0.87; s-MLPED—LogMAR BCVA: Baseline = 0.43 [∼ 20/54] ± 0.68; Final = 0.94 [∼ 20/174] ± 0.71; *p* = 0.06). Moreover, after presentation, the median number of injections in DN-MLPED eyes were significantly lower compared to s-MLPED eyes (DN-MLPED:4; s-MLPED:12; *p* = 0.03) (Median follow-up: DN-MLPED = 26 months; s-MLPED = 54 months; *p* = 0.15). Subretinal hyperreflective-material (SHRM) deposition heralded the onset of multilayering and was seen to progress in all DN-PED eyes and 1/4 eyes of s-MLPED. To conclude, MLPED is a unique form of cicatrizing fibrovascular-PED which can evolve denovo too. Long-standing disease with intermittent or low-grade activity can potentially explain this unique phenomenon. With fewer anti-VEGF therapy, the de-novo MLPED eyes show more visual stability as compared to s-MLPED eyes.

## Introduction

Spectral-domain optical coherence tomography (SD-OCT) has revolutionized our ability to image the sub-retinal pigment epithelium (RPE) space in the eyes with pigment epithelial detachment (PED). A detailed analysis of the sub-RPE compartment is now possible with high-resolution SD-OCT images that are comparable with histopathological grade specimens^1–5^. A distinctive feature associated with fibrovascular PEDs, termed as multilayered PED has been reported in neovascular age-related macular degeneration (n-AMD) eyes^6^. The multilayering was noted as a response to chronic anti-vascular endothelial growth factor (anti-VEGF) therapy in these eyes^7^. Various features of MLPED have been previously described in the literature such as sub-RPE material^7^ and prechoroidal clefts^6^. Theories of the pathogenesis of multi-layered PED have been put forth. It has been proposed that fibrinous exudation from the overlying choroidal neovascular (CNV) complex precipitates on the surface of Bruch's membrane with the remaining fibrin serving as a scaffold for vascular and connective tissue deposition^6^. Major significance has been given to the role of chronic anti-VEGF pharmacotherapy which stabilizes and causes organization of the neovascular process within the sub-RPE space, resulting in preservation of the overlying RPE and photoreceptor layer^6^.

Despite the current knowledge, many aspects of MLPED remain unexplored, such as its prevalence, occurrence of this phenomenon in non n-AMD neovascularization, underlying developmental factors other than anti-VEGF therapy, and so on. This study is an attempt in this direction and aims to describe evolutionary variants of MLPED especially those occurring in the absence of anti-VEGF therapy, which we designate as de-novo MLPED (DN-MLPED). We analyze their SD-OCT features, treatment profile, and visual outcomes and compare them with similar characteristics of secondary MLPED (s-MLPED) which occur following chronic anti-VEGF therapy. Furthermore, we uniquely describe the occurrence of MLPED in association with polypoidal choroidal vasculopathy (PCV).

## Methods

This study was a retrospective analysis of eyes with CNV who presented/developed features of MLPED during the follow-up. The study was conducted in accordance to the tenets of the Declaration of Helsinki and was approved by the Institutional Review Board of Chaithanya Eye Hospital and Research Institute, Trivandrum, India. Written informed consent was obtained from all the participants. All eyes with a diagnosis of MLPED with a minimum follow up of 12 months were included in the study. All patients underwent routine ophthalmic examination and serial evaluation including fundus fluorescein angiography (FFA), indocyanine green angiography (ICGA), and SD-OCT on Spectralis HRA + OCT (Heidelberg Engineering, Heidelberg, Germany).

204 eyes of patients with CNVM were analysed and eyes with features of multilayering were included in the study. Diagnosis of MLPED was established with a cross-sectional SD-OCT scan by a single masked observer (MS). Diagnosis of MLPED was based on the primary description of this entity by Rahimy et al. i.e., the presence of fusiform, or spindle-shaped, complex of highly organized, layered, homogenous reflective bands within the PED^6^. Though multilayering was the definitive feature, initiation of MLPED was described when there was a minimum of at least 2 lamellae within the PED. Progressive growth of the multilayering in the PED was mapped at each follow-up visit in such cases. History of treatment with anti-VEGF injections was noted and the eyes with a positive history in presence of MLPED were diagnosed as secondary MLPED (s-MLPED). In the eyes where MLPED developed in the absence of anti-VEGF treatment history were labelled as de-novo MLPEDs. Systemic associations were recorded. Uncontrolled hypertension was diagnosed based on JNC Guidelines^8^.

Based on FFA and ICGA, the eyes were diagnosed as typical n-AMD or PCV. Baseline SD-OCT features, including the nature of multilayering, presence of hyporeflective choroidal clefts, and associated overlying RPE and intraretinal features were evaluated. Multilayering spindles within the PED were classified based on its reflectivity (with outer plexiform layer as the reference) as hyper-, hypo- or isoreflective and based on the intrinsic pattern as compact or loose. The overlying RPE in these MLPEDs were classified as bumpy pattern with uneven RPE contour changes, crenated pattern with fine RPE serrations, focal RPE breaks and popcorn pattern having herniation of possible neovascular tissue through a presumed break of the RPE. The presence of hyporeflective intraretinal and choroidal clefts and the change in the nature of the clefts were analysed. Other OCT features that immediately preceded the event of multilayering in these eyes were also analysed.

Based on the underlying diagnosis of n-AMD or PCV, these PED’s were treated with intravitreal anti-VEGF monotherapy or in combination with photodynamic therapy (PDT). The treatment modality, dosing regimen, and frequency of intravitreal anti-VEGF injections were determined by the treating physician. Correlation with treatment protocols and visual acuity (VA) was done. Eyes with DN-MLPED and s-MLPED were compared based on the SD-OCT features, treatment profile, and VA outcomes.

For purposes of statistical analysis, all Snellen visual acuity data were converted to LogMAR values. Continuous variables were described as mean and variation of each observation from the mean value (Standard deviation) represented as mean ± SD or median and interquartile range if they failed to follow a normal distribution. Differences between the groups were tested for statistical significance with unpaired t-tests for normal distribution and using Mann Whitney U test for non-normal distribution. The differences during follow-up within each group were compared with paired *t* tests. *p* value < 0.05 was considered statistically significant.

## Results

Out of the 204 eyes which were analysed, 17 eyes (8.3%) of 16 patients were identified to have MLPED features. There were 7 females and 9 males respectively with a mean age of 74 ± 5.36 years. Table 1 provides patient demographic and ocular findings.

**Table 1:** Baseline patient profile, systemic findings, and angiographic results.

Variable	Observation
**Gender**	
Males	9 patients
Females	7 patients
**Mean age**	74 ± 5.36 years
**Systemic comorbidities**	
Hypertension	10 patients
Diabetes	8 patients
Cardiac disease	5 patients
Cancer	3 patients
Parkinson’s	1 patient
Hepatitis	2 patients
**FFA findings**	
Occult	15 (88.2%)
Mixed	2 (11.7%)
Classic	0
**ICGA diagnosis**	
n-AMD	7 (41.1%)
PCV	10 (58.82%)
**Mean LogMAR BCVA at Baseline**	0.58 (∼ 20/76) ± 0.82

FFA: Fundus fluoresceine angiography; ICGA: Indocyanine green angiography; n-AMD: Neovascular age-related macular degeneration; PCV: Polypoidal choroidal vasculopathy; BCVA: Best corrected visual acuity; CFCF: Counting finger close to face.

Of these, 17 eyes 7 eyes (41.18%) had DN-MLPED while 10 eyes (58.82%) had s-MLPED, respectively. There was no significant difference in age between the DN-MLPED and s-MLPED eyes (DN-MLPED: 71.71 ± 4.68 years; s-MLPED: 75.5 ± 5.44 years; *p* = 0.07). Table 2 provides comparative data between DN-MLPED and s-MLPED.

**Table 2:** Comparison between de-novo MLPED and s-MLPED features.

	De-novo MLPED	s-MLPED	*p* value
No of eyes	7	10	
Age	71.71 ± 4.68	75.5 ± 5.44	0.07
n-AMD	4	3	
PCV	3	7	
Type of CNVM	Occult 5; Mixed 2	Occult 10	
Mean baseline BCVA	0.79 (∼ 20/123) ± 0.91	0.43 (∼ 20/54) ± 0.68	0.2
Mean final BCVA	0.76 (∼ 20/115) ± 0.73	0.94 (∼ 20/174) ± 0.71	0.31

	Change in BCVA *p* = 0.87	Change in BCVA *p* = 0.06	
SFCT	194.14 ± 71.66	217.44 ± 76.96	0.27
Mean injections	6 ± 5.1	16.3 ± 12.68	0.03*
Median injections	4	12	0.03*
Mean follow-up (months)	30.64 ± 25.28	52 ± 27.64	0.06
Median follow-up (months)	26	54	0.15
ML reflectivity	Iso Compact–3	Iso Compact–5	
	Iso Loose–2	Iso Loose–3	
	Hypo Compact–1	Hyper Compact–2	
	Hyper Compact—1		
Choroidal cleft	3	4	
Intraretinal cleft	1	1	
SHRM	3; All had increased activity	4; Only 1 had increased activity	

MLPED: multilayered pigment epithelial detachment; s-MLPED: secondary multilayered pigment epithelial detachment; n-AMD: Neovascular age-related macular degeneration; PCV: Polypoidal choroidal vasculopathy; CNVM: Choroidal neovascular membrane; BCVA: Best corrected visual acuity; SFCT: Sub-foveal choroidal thickness; ML: Multilayer; SHRM: Subretinal hyperreflective material; CFCF: Counting finger close to face; CF: Counting fingers.

*Statistically significant.

The mean follow-up period was 43.24 ± 28 months, and there was no significant difference in the median follow-up between the DN-MLPED and s-MLPED eyes (DN-MLPED: 26 months; s-MLPED: 54 months; *p* = 0.15). Ten of these 16 patients had uncontrolled hypertension, 4 belonging to the DN-MLPED group, and 6 to the s-MLPED group. Additional clinical data collected for each patient included systemic comorbidities as shown in Table 1.

The mean LogMAR best-corrected visual acuity (BCVA) was 0.58 (∼ 20/76) ± 0.82 at the time of diagnosis of MLPED which decreased to 0.86 (∼ 20/145) ± 0.71 at the final visit. However, this difference was not statistically significant (*p* = 0.1). On subgroup analysis, there was no difference in VA between the two groups at baseline (LogMAR BCVA = DN-MLPED: 0.79 (∼ 20/123) ± 0.91; s-MLPED: 0.43 (∼ 20/54) ± 0.68; *p* = 0.2) and at the final visit (LogMAR BCVA = DN-MLPED: 0.76 (∼ 20/115) ± 0.73; s-MLPED: 0.94 (∼ 20/174) ± 0.71; *p* = 0.31) respectively. However, in the DN-MLPED eyes, the mean LogMAR BCVA improved (Baseline visit: 0.79 (∼ 20/123) ± 0.91; Final visit: 0.76 (∼ 20/115) ± 0.73; *p* = 0.87) while it reduced in the s-MLPED eyes (Baseline visit: 0.43 (∼ 20/54) ± 0.68; Final visit: 0.94 (∼ 20/174) ± 0.71; *p* = 0.06), although this difference was not significant.

**Figure 1:**
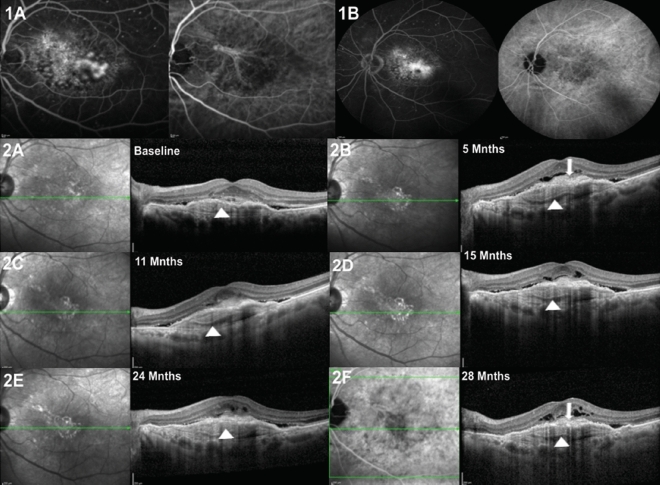
Left eye of a 65-year-old male with neovascular age-related macular degeneration (nAMD), and denovo multilayered pigment epithelial detachment (DN-MLPED). Fundus fluorescein angiography (FFA) shows occult choroidal neovascular membrane (CNVM) with a classic component and a well-defined neovascular network with feeder vessel on indocyanine green angiography (ICGA; **A**). Serial cross-sectional optical coherence tomography (OCT) images over 28 months demonstrate the onset and persistence of subretinal hyperreflective material (SHRM; arrows) along with multilayering (Arrowheads) (**A**–**F**).

**Figure 2:**
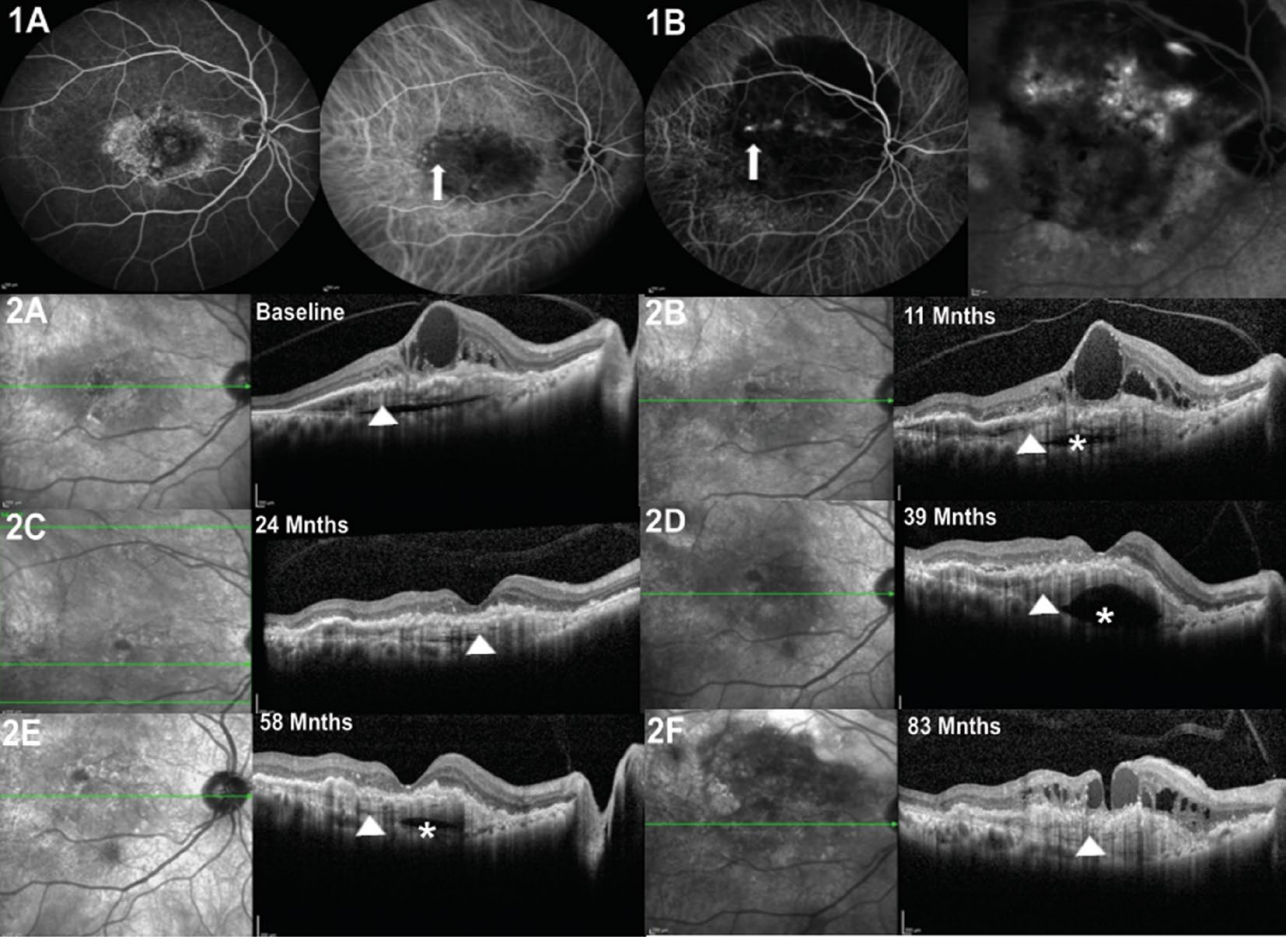
Right eye of a 79-year-old female with the occurrence of polypoidal choroidal vasculopathy (PCV) and de novo multilayered pigment epithelial detachment (DN-MLPED). Angiography (**A**) shows a neovascular network with polyps (arrow), more evident on follow up indocyanine green angiography (ICGA; **B**). Serial cross-sectional optical coherence tomography (OCT) over 83 months shows increasing multilayering under the retinal pigment epithelium (RPE; arrowhead) with subretinal hyperreflective material (SHRM; Red *) (**A**–**F**). The waxing and waning nature of choroidal cleft is seen (White *).

**Figure 3:**
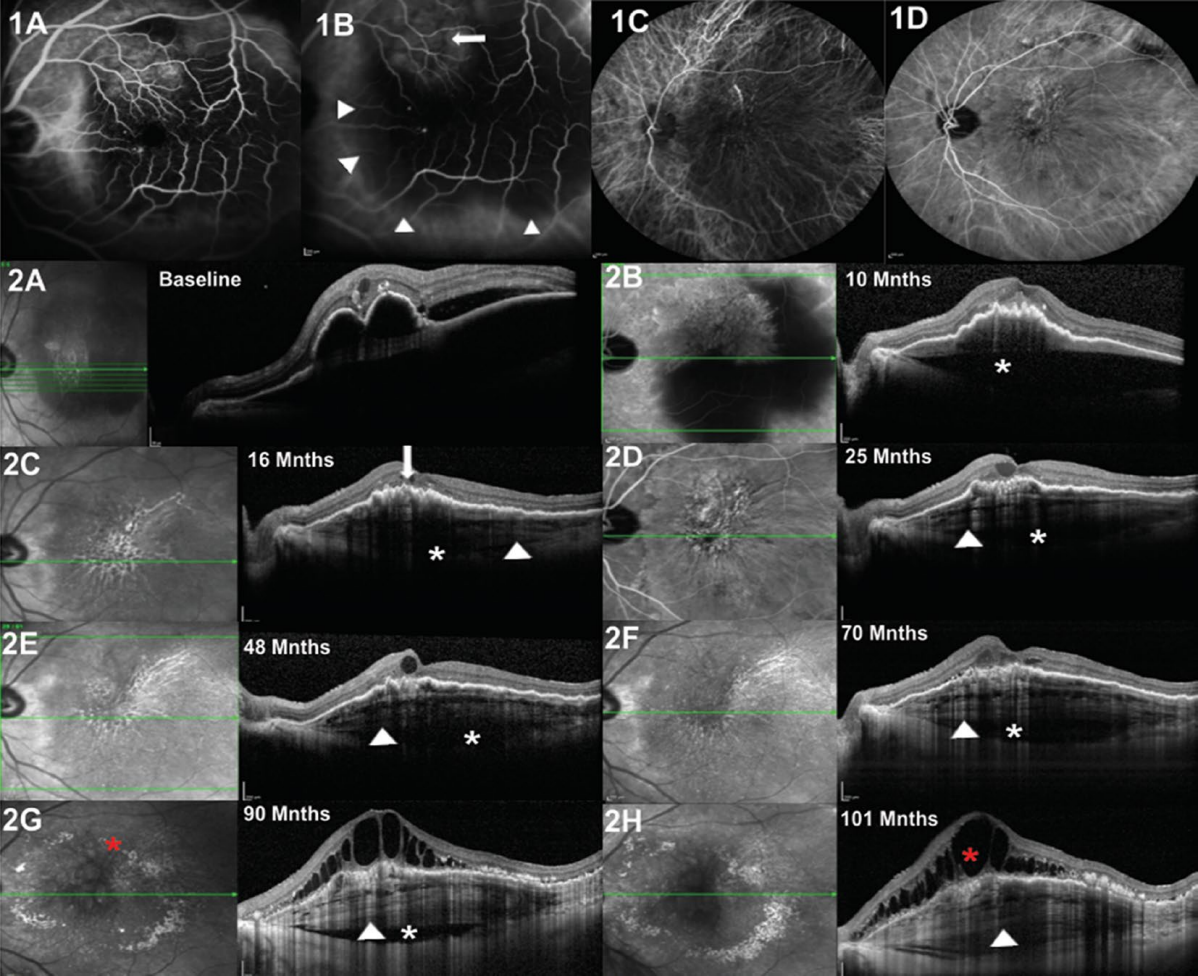
Left eye of an 83-year-old male with age-related macular degeneration (nAMD) and multilayered pigment epithelial detachment secondary to chronic anti-vascular endothelial growth factor (anti-VEGF) therapy (s-MLPED). Baseline angiography shows a neovascular network (arrow) with large pigment epithelial detachment (PED; arrowheads) (**B**). An increase in the size of the network was noted on follow-up indocyanine green angiography (ICGA; **D**). Serial cross-sectional optical coherence tomography (OCT) over a period of 101 months shows an increase in multilayering (arrowhead) with a persistent choroidal cleft (white*) and irregular crenated retinal pigment epithelium (RPE; arrow; **A**–**H**). The onset of Multilayering was seen at 16 months after starting anti-VEGF therapy (**C**) which continued to increase during treatment (**D**–**H**). Persistent intraretinal fluid and exudation (red*) can be seen late during follow up (**G**, **H**).

**Figure 4:**
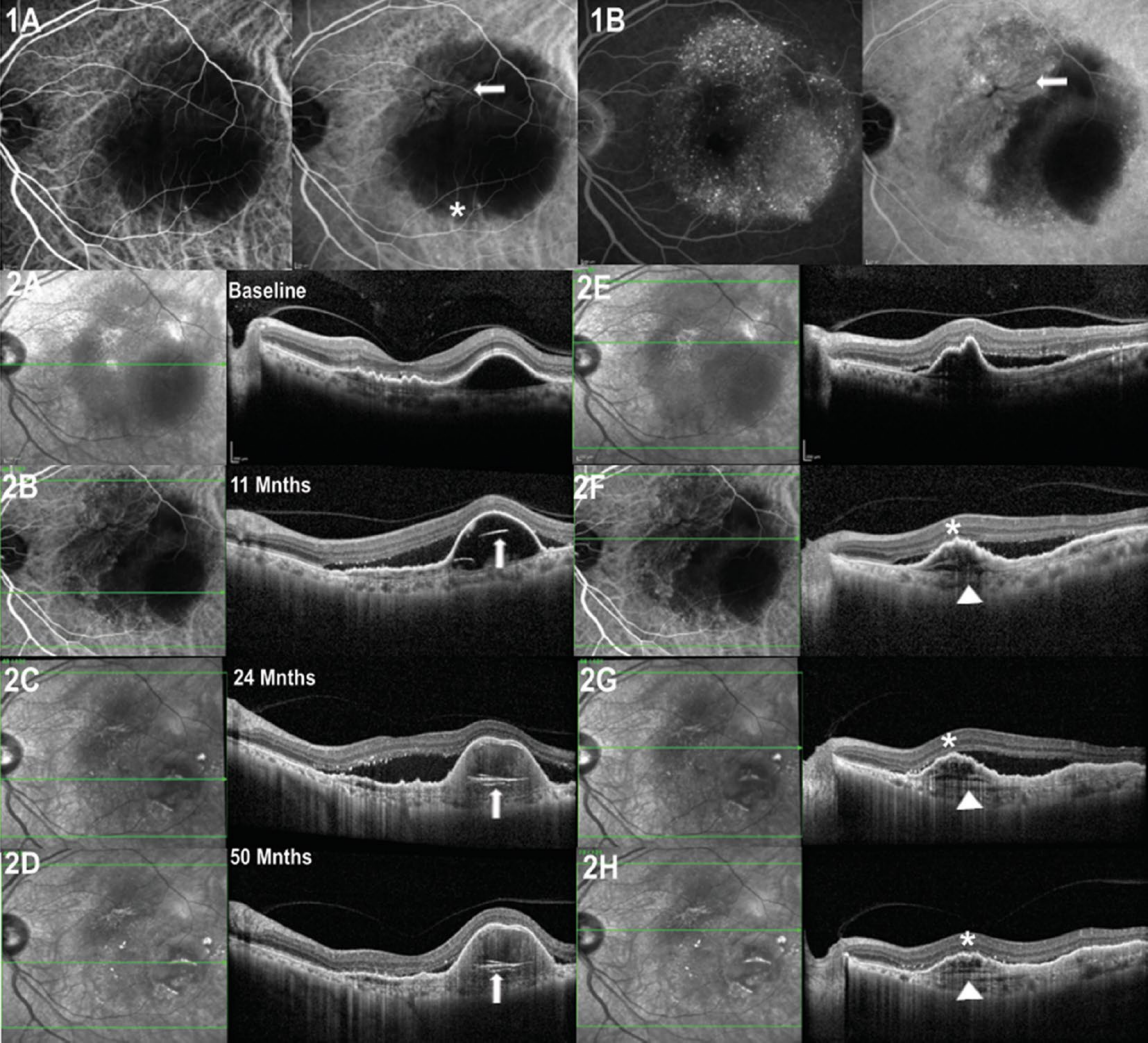
Left eye of a 77-year-old male with polypoidal choroidal vasculopathy (PCV) and multilayered pigment epithelial detachment secondary to chronic anti-VEGF therapy (s-MLPED). Baseline indocyanine green angiography (ICGA) shows a polyp (*) with a neovascular network (arrow) and large pigment epithelial detachment (PED; **A**). Follow-up ICGA shows an increase in the size of the neovascular network arrow) (**B**). Serial cross-sectional optical coherence tomography (OCT) over a period of 50 months shows increasing multilayering (arrowheads) with irregular crenated retinal pigment epithelium (RPE; *) in the superior portion of PED (**F–H**) and concurrent occurrence and increase of hyperreflective laminated bodies (arrows) in the temporal portion of PED (**B–D**). Note the absence of choroidal cleft.

The underlying ocular disease associated with the MLPED was n-AMD in 7 eyes (41.18%) and PCV in 10 eyes (58.82%) based on multimodal imaging (Table 1). In the DN-MLPED eyes, 3 eyes had PCV (42.86%) while 4 eyes had n-AMD (57.14%) whereas in s-MLPED eyes, 7 eyes had PCV (70%) and 3 eyes had n-AMD (30%), respectively. Case examples demonstrating the occurrence of DN-MLPED in AMD and PCV eyes and s-MLPED in AMD and PCV eyes are illustrated in Figs. 1, 2, 3, and 4, respectively. On FFA, 15 eyes were classified as occult CNVM and 2 eyes had mixed CNVM (Table 1). The 2 eyes with mixed CNVM demonstrated DN-MLPED while all the 10 eyes with s-MLPED had occult leakage on FFA. On SD-OCT, isoreflectivity with compact arrangement of layers was most frequently encountered, both in the DN-MLPED eyes (3/7) and s-MLPED eyes (5/10) (Table 2).

Intraretinal cleft was noted in 2 eyes, one each in the DN-MLPED and s-MLPED group (Tables 2, 3), and showed a decrease in DN-MLPED eye during treatment. Recurring and disappearing choroidal clefts were seen in 7 eyes (DN-MLPED: 3 eyes; s-MLPED: 4 eyes) (Tables 2, 3). Subretinal hyperreflective material (SHRM) was noticed in 7 eyes (DN-MLPED: 3 eyes; s-MLPED: 4 eyes), 4 eyes demonstrated increased SHRM activity with the onset of multilayering including all the DN-PED eyes and 1/4^th^ of s-MLPED eyes (Tables 2, 3). In relation to the overlying RPE abnormalities, RPE crenations were most seen (70.58%) followed by bumpy RPE (29.41%), popcorn lesions (5.88%) and focal breaks (5.88%). Additional subgroup data regarding the SD-OCT features are presented in Table 3.

**Table 3:** Spectral domain optical coherence tomography findings in MLPED.

SD-OCT finding	Eyes (%)
**Overlying RPE abnormality**	
Crenation	12 (70.58)
Bumpy	5 (29.41)
Popcorn lesion	1 (5.88)
Focal breaks	1 (5.88)
**Neovascular tissue under RPE**	
Altered Reflectivity/Diffuse	10 (58.82)
Organized	7 (41.17)
**Choroidal Cleft**	7 (41.17)
**Intraretinal Cleft**	2 (11.76)
**SHRM presence**	7 (41.17)
Increasing SHRM	4 (17.64)

MLPED: multilayered pigment epithelial detachment; SD-OCT: Spectral Domain Optical Coherence Tomography; RPE: Retinal pigment epithelium; Crenated RPE seen as undulated Microfolds of RPE; Popcorn lesion refers to focal reflective lesion at the RPE simulating herniation of neovascular tissue breaching RLE, Focal breaks seen as punctate defects in the RPE; SHRM: Subretinal hyperreflective material.

Six eyes with PCV underwent combination therapy with PDT and anti-VEGF injections while four PCV eyes and all seven eyes of n-AMD underwent anti-VEGF monotherapy. All intravitreal anti-VEGF injections were given as three monthly loading doses followed by a PRN regime. The details of the treatment are provided in Table 4. The median number of injections at the final visit in all MLPED eyes was 9 (range 1–41 injections). On subgroup analysis, the median number of injections in the DN-MLPED eyes (4 injections [range 1–16 injections]) were significantly lower as compared to s-MLPED eyes (12 injections [range 4–42 injections]). Amongst the DN-MLPED eyes, the mean number of injections were significantly more in eyes with PCV as compared to nAMD (PCV: 10.33 ± 4.19; nAMD: 2.75 ± 1.09; *p* = 0.02). However, in the s-MLPED arm, no significant difference in median injections was between the PCV (15 [range 7–16 injections]) and nAMD (9 [range 4–42 injections]) eyes (*p* = 0.49). Based on the FFA pattern, no significant difference was noted in the median number of injections between the eyes with occult CNVM (6 [range 1–16 injections]) and mixed CNVM (3.5 [range 3–4 injections]) (*p* = 0.14).

**Table 4:** Treatment of MLPED eyes.

Treatment	Eyes (%)
**PDT**	6 (35.29%)
**Intravitreal pharmacotherapy**	
Ranibizumab	7 (41.17)
Aflibercept	1 (5.88)
Bevacizumab	0 (0)
Ranibizumab + Aflibercept	4 (23.5)
Ranibizumab + Bevacizumab	2 (11.76)
Ranibizumab + Bevacizumab + Aflibercept	3 (17.64)

MLPED: multilayered pigment epithelial detachment; PDT: Photodynamic therapy.

## Discussion

The detection of a layer of tissue behind the RPE in PEDs associated with occult CNV was first made by Coscas et al^9^. Layers or lamella with clefts within fibrovascular PEDs were first identified by Spaide in 10 eyes with n-AMD that underwent enhanced depth imaging SD-OCT^7^. Rahimy et al. further defined Multilayered PED as a characteristic fusiform complex of highly organized, layered, hyperreflective bands in eyes with n-AMD receiving serial intravitreal anti-VEGF injections and proposed its pathogenesis^6^. This is the largest series of this pathology available in the literature and included all eyes of n-AMD while multi-layered phenotype described by Adrian Au et al. also involved similar n-AMD patients^6,10^. In the current study, classification of CNVM based on angiography revealed 15 eyes of occult CNVM and 2 eyes of mixed CNVM. In comparison, all 22 eyes analysed by Spaide and Adrian Au et al. had occult CNVM^7,10^. Also, in our series, the underlying ocular disease associated with the MLPED was PCV in 10 eyes and neovascular AMD in 7 eyes. This is the first series to highlight the occurrence of MLPED features in PCV eyes. PCV represents part of the pachychoroid disease spectrum in which type 1 CNVM develops secondary to chronic choroidal thickening and/or focal pachyvessels with overlying choriocapillaris loss^11,12^. This spectrum is known to have periods of disease activity followed by dormancy. Earlier the 'Triple-layer sign' has been described in eyes with PCV, representing sub-RPE neovascular tissue, hyporeflective space, and underlying reflective choroid^13^. We believe that multilayering is a unique characteristic manifestation of smouldering disease activity and may represent persistent subclinical active disease. Moreover, characteristic, resistant branching vascular membranes (BVN) have been reported in Everest and Planet studies where long-term anti-VEGF therapy and/or PDT was adopted^14,15^. In our series, MLPED was more common in PCV eyes (58.82%) as compared to n-AMD (41.18%). Future prospective studies with video-based ICGA would provide valuable insight into the association of MLPED, both de-novo and secondary, with a BVN.

MLPED is an infrequent form of PED and was seen in only 8.3% of the study eyes. Contrary to the report by Rahimy et al. we found that MLPED could arise denovo in the absence of chronic anti-VEGF^6^. In fact, 41.18% of the MLPEDs had de-novo origin while 58.82% of them were secondary to anti-VEGF therapy. There can be few theories for such de-novo nature of MLPED evolution. Intermittent deturgescence during phases of disease activity can potentially be one such theory. Against this background, we observed varied forms of slackness of the MLPED surface, such as crenations, bumpiness, and even focal breaks or popcorn lesions. Periodical deturgescence can probably lead to some kind of cyclical deflation or abridgment of the PED and its content causing multilayering. Another possible reason could be the long-standing nature of the disease which can precipitate intermittent deposition of exudative material during the active period. Such as the occurrence of denovo MLPED has not been described in the literature.

Features of MLPED described by Rahimy et al. include organized hyperreflective bands and Prechoroidal clefts^6^, which were seen in our series also. This observation supports the view of Rahimy et al.^6^ that this could be fibrinous exudate with contractile properties from under the neovascular tissue and deposited over the Bruch’s membrane. We could also demonstrate that with chronicity, the bands show increased reflectivity and layering probably due to increased fibrous tissue or fibrinous condensation, or both. Also, additional hyperreflective laminar deposits were noted in 3 eyes as described by Clements et al. and these could be a manifestation of organized fibrin deposits or represent detached neovascular tissue^16^. Similar hyperreflective crystalline deposits (HCDs) in the subretinal pigment epithelium–basal laminar space has also been described by Fraggiota et al^17^. In the series by Rahimy et al.^6^, the MLPED reflectivity was evaluated and was noted to be hyperreflective in all of them. In contrast, we observed isoreflectivity of the layering to be most common, accounting for half of the s-MLPED cases and 42.86% of DN-MLPED cases. Additionally, these eyes also demonstrated compact arrangement of the layering. The variable reflectivity and compactness of the MLPED layering can be secondary to the fluctuating course of neovascularization and cicatrization of the underlying tissue. Further histopathological studies are warranted to further explore these tomographic findings.

Choroidal Clefts were noted in 7 patients during the follow-up period. Choroidal clefts are believed to occur either due to fluid accumulation due to disease activity and located below the neovascular element in a fibrovascular PED or due to mechanical separation of the fibrous tissue from the underlying choroidal vasculature^6^. In our series, all cases with choroidal clefts showed reduction after anti-VEGF therapy, only to recur with new disease activity. The stability of intraretinal cleft was noted in 2 eyes probably representing degenerative cysts.

Rahimy et al. have shown that MLPED eyes maintain good visual acuity^6^. However, in our series, the MLPED eyes showed a trend towards VA reduction, over a median follow-up of almost three years (32 months). On subgroup analysis, eyes having a de-novo variant of MLPED showed a trend towards improvement in vision while the secondary variant of MLPED resulting post anti-VEGF treatment demonstrated decline in vision. This visual decline seen in secondary MLPED eyes, in contrast to an improvement in VA demonstrated by Rahimy et al.^6^, could be related to the ethnic differences between the patient populations, inadequate treatment, or presence of underlying PCV/BVN (7/10 s-MLPED eyes in our series), which are known to be resistant to anti-VEGF therapy. Nonetheless, eyes with DN-MLPED demonstrated some improvement in VA. This is a very interesting observation and can be related to a more stable neovascular tissue complex as compared to s-MLPED eyes. This can also be gauged from the fact that the median number of injections in the de-novo eyes were significantly lower as compared to the secondary MLPED eyes. Although on subgroup analysis, the median injections were fewer in nAMD eyes as compared to PCV eyes in the de-novo group, the small number of eyes may be inadequate to draw notable conclusions. Nonetheless, it does suggest a possible need for frequent injections in PCV eyes with DN-MLPED. Additionally, the larger size of standard deviation in the mean number of injections for PCV eyes (± 13.85) in the s-MLPED group may possibly confound the comparison with nAMD eyes, and it is unknown whether this causes the lack of significance in the comparison, and whether a much larger sample size would show a significant difference.

The occurrence of multilayering even before starting the anti-VEGF treatment is suggestive of early cicatrization and compartmentalization of the neovascular tissue complex in the sub-RPE space. Such a confined tissue complex may provide adequate oxygenation and nourishment to the outer retinal layers, thereby maintaining their functionality and consequently retaining good visual acuity. Additionally, it is plausible that DN-MLPED contains a relatively inactive CNV complex supported by the fact that these eyes maintained good visual acuity with a fewer number of injections. This incomplete cicatrization of PEDs thus enable better stability and maintain relatively good vision in these eyes.

SHRM showed progression in 4 eyes and it seemed to herald the multilayering pattern in these PEDs. These probably could indicate subclinical type 2 disease activity as also evidenced by the observation of mixed CNVM on FFA in some of the eyes. It could be speculated that when this tissue contracts, the underlying RPE crenations appear with inadvertent breaks and popcorn lesions in the RPE with subsequent deturgescence of the large PED. This may modify the nature and response of the underlying neovascular tissue manifesting as multilayering at the base of the PED. Interestingly, SHRM was seen in 3 out of the 7 eyes with DN-MLPED, and remarkably it progressed in all of them. The precise characterization of SHRM remains a perplexing question, but the literature suggests its composition to be a mixture of exudation, fibrosis, neovascular tissue, and hemorrhage^18^. Each of these components has been associated with the phenomenon of multilayering as they represent periods of activity and inactivity. Hence, the relationship of concurrent progression of SHRM and MLPED is indicative of analogous pathogenesis and similar composition of materials.

The current study is the first to report the occurrence of denovo MLPED and its presence in PCV eyes. We speculate that anti-VEGF may not be the only implicating event in these eyes and believe that multilayering may just be a manifestation of a chronic self-cicatrizing disease process. Patients with DN-MLPED probably did not report a long history because they had mild visual disturbances which could easily be missed by the patient, especially if unilateral. Alternatively, the process of multilayering may be a natural process observed in some patients with fibrovascular PED with some genetic predisposition or influence of coexistent systemic disease. We observed that 10 of the 16 patients had uncontrolled hypertension in our series. It is well known that hypertension is a known association in Asian eyes manifesting active PCV^19,20^. A similar phenomenon may exist in these eyes too where there is transudation rather than exudation from the neovascular tissue in these hypertensive patients which could explain the reflective nature of the deposits. A detailed and larger series may yield more answers and improve our understanding of this curious pathology.

The limitation of the study includes the lack of a control group, limited sample size, lack of standardized BCVA measurement and use of varied treatment modalities such as single and mixed anti-VEGF treatments, as well as PDT or combination of the above, which can introduce known and unknown confounding effects that reduces the reliability of correlating the numbers of injections with various lesion subtypes. Inclusion of a control arm including eyes with fibrovascular PEDs receiving multiple intravitreal injections and not manifesting multilayering would have added credence to the observations, inferences, and hypothesis. Furthermore, a larger sample size with standardized treatment regimen is critical to validate the results of our study.

To conclude, MLPED is a unique form of fibrovascular PED which can occur in denovo. The underlying cause can be either nAMD or PCV. DN-MLPED eyes maintain reasonable vision with fewer injections as compared to MLPED secondary to chronic anti-VEGF therapy. The presence of SHRM was associated with the start of multilayering and might be a significant indicator of this pathological process.

## Data Availability

The datasets generated during and/or analysed during the current study are available from the corresponding author on reasonable request.
